# Coronary flow reserve evaluated by phase-contrast cine cardiovascular magnetic resonance imaging of coronary sinus: a meta-analysis

**DOI:** 10.1186/s12968-023-00912-5

**Published:** 2023-02-20

**Authors:** Toshiki Tanigaki, Shingo Kato, Mai Azuma, Masanori Ito, Nobuyuki Horita, Daisuke Utsunomiya

**Affiliations:** 1 Department of Diagnostic Radiology, Yokohama City University Graduate School of Medicine, Yokohama, Japan; 2 Department of Cardiology, Kanagawa Cardiovascular and Respiratory Center, Yokohama, Japan; 3 Department of Diagnostic Radiology, Kanagawa Cardiovascular and Respiratory Center, Yokohama, Japan; 4 Chemotherapy Center, Yokohama City University Graduate School of Medicine, Yokohama, Japan

**Keywords:** Coronary flow reserve, Coronary sinus, Coronary artery disease, Phase-contrast, Meta-analysis

## Abstract

**Background:**

Phase-contrast cine cardiovascular magnetic resonance (CMR) of the coronary sinus has emerged as a non-invasive method for measuring coronary sinus blood flow and coronary flow reserve (CFR). However, its clinical utility has not yet been established. Here we performed a meta-analysis to clarify the clinical value of CMR-derived CFR in various cardiovascular diseases.

**Methods:**

An electronic database search was performed of PubMed, Web of Science Core Collection, Cochrane Advanced Search, and EMBASE. We compared the CMR-derived CFR of various cardiovascular diseases (stable coronary artery disease [CAD], hypertrophic cardiomyopathy [HCM], dilated cardiomyopathy [DCM]) and control subjects. We assessed the prognostic value of CMR-derived CFR for predicting major adverse cardiac events (MACE) in patients with stable CAD.

**Results:**

A total of 47 eligible studies were identified. The pooled CFR from our meta-analysis was 3.48 (95% confidence interval [CI], 2.98–3.98) in control subjects, 2.50 (95% CI, 2.38–2.61) in stable CAD, 2.01 (95% CI, 1.70–2.32) in cardiomyopathies (HCM and DCM). The meta-analysis showed that CFR was significantly reduced in stable CAD (mean difference [MD] = −1.48; 95% CI, −1.78 to −1.17; p < 0.001; I^2^ = 0%; p for heterogeneity = 0.33), HCM (MD = −1.20; 95% CI, −1.63 to −0.77; p < 0.001; I^2^ = 0%; p for heterogeneity = 0.49), and DCM (MD = −1.53; 95% CI, −1.93 to −1.13; p < 0.001; I^2^ = 0%; p for heterogeneity = 0.45). CMR-derived CFR was an independent predictor of MACE for patients with stable CAD (hazard ratio = 0.52 per unit increase; 95% CI, 0.37–0.73; p < 0.001; I^2^ = 84%, p for heterogeneity < 0.001).

**Conclusions:**

CMR-derived CFR was significantly decreased in cardiovascular diseases, and a decreased CFR was associated with a higher occurrence of MACE in patients with stable CAD. These results suggest that CMR-derived CFR has potential for the pathological evaluation of stable CAD, cardiomyopathy, and risk stratification in CAD.

**Supplementary Information:**

The online version contains supplementary material available at 10.1186/s12968-023-00912-5.

## Introduction

Evaluating the microcirculation is extremely important in the diagnosis of coronary artery disease (CAD). A new disease concept, ischemic nonobstructive CAD (INOCA), was proposed to describe a condition in which myocardial ischemia occurs despite the absence of an obstructive lesion in the epicardial coronary artery. American Heart Association/American College of Cardiology guidelines also emphasize the importance of microcirculatory disturbances in INOCA. Moreover, microcirculatory disturbances are involved in the pathogenesis of various cardiovascular diseases [1]. The prevalence of microvascular dysfunction (MVD) is higher than ever in many clinical settings [2, 3], and its presence is associated with worse clinical outcomes [4]. Various indices have been proposed to evaluate microcirculatory disturbances, one of which is coronary flow reserve (CFR). CFR is an index of a combination of the epicardial coronary artery and the microvasculature [5]. In the absence of epicardial CAD, impairment of the CFR suggests the presence of MVD. Positron emission tomography (PET) is an established non-imaging modality for evaluating CFR [6]. However, PET imaging has some limitations, including radiation exposure, availability, and high cost. These disadvantages limit the widespread clinical use of PET for assessing CFR.

Phase-contrast cine cardiovascular magnetic resonance (CMR) of the coronary sinus is another method used to quantify myocardial blood flow (MBF) [7]. An estimated 96% of the blood flow is returned through the myocardium through the coronary sinus. Therefore, coronary sinus blood flow can approximate the total MBF. The volume of myocardial blood flow per gram of myocardium can be estimated by dividing the coronary sinus blood flow by the myocardial weight. The ratio of MBF can be used to calculate CFR during pharmacological stress divided by that at rest, which is linearly correlated with PET-derived CFR [7]. Owing to its ability to test without radiation exposure, CMR-derived CFR overcomes the limitations of PET-derived CFR. However, owing to limited evidence, the clinical relevance of CMR-derived CFR is not well known. Therefore, here we performed a meta-analysis to compare CMR-derived CFR findings of patients with cardiovascular diseases and healthy controls. We also assessed the prognostic value of CMR-derived CFR in patients with stable CAD.

## Materials and methods

### Literature search

We searched the electronic databases PubMed, Web of Science Core Collection, Cochrane Advanced Search, and EMBASE using the search formulas listed in the Appendix (Additional file 1). We performed the database search on May 25, 2022. After screening all abstracts from the search results, potentially relevant studies were reviewed by two reviewers (SK and MA) for final eligibility. A third reviewer resolved disagreements between them. This meta-analysis adhered to the Preferred Reporting Items for Systematic Reviews and Meta-Analyses guidelines. This protocol was registered in the University Medical Informatics Network (R000054826). We did not obtain institutional review board approval since this study was a meta-analysis.

### Eligibility criteria and outcomes

We included prospective and retrospective studies that included data on CFR evaluated by phase-contrast CMR of the coronary sinus in patients with cardiovascular diseases and CAD risk factors. The subjects included healthy individuals and controls (a group of patients who underwent CMR for some clinical reason but in whom no abnormalities were detected), those with CAD, and those with cardiomyopathy (hypertrophic cardiomyopathy [HCM] and dilated cardiomyopathy [DCM]). Studies of other diseases were excluded from the meta-analysis. We did not include studies on myocardial perfusion reserve using stress-perfusion CMR, as the methodology is completely different. Only articles published in English were included in this study.

Two reviewers extracted the study characteristics, including author name, publication year, country of origin, patients’ diseases, and CMR parameters (SK, MA). First, the disease, age, sex, and CMR parameters for each study were extracted (in addition to CFR from phase-contrast cine CMR, which was the focus of the study, MBF calculated from blood flow in the coronary sinus, left ventricular (LV) function, LV volume, and late gadolinium enhancement (LGE). Second, we compared the CFR between cardiovascular disorders and healthy controls. Finally, the hazard ratio (HR) of CFR for the major adverse cardiac event (MACE) was evaluated in patients with CAD.

In the literature, two separate methods are used to calculate CFR. The first method calculates the CFR by dividing the blood flow in the coronary sinus at stress by the blood flow at rest. The second method is to divide the coronary sinus blood flow by the LV myocardial weight to calculate the MBF (mL/min/g) and then divide the MBF at stress by the MBF at rest to calculate CFR. Because the CFR values are the same for both methods, those calculated by these two methods are harmonized. We also used the Newcastle–Ottawa Quality Assessment Scale and Case Control Studies to evaluate the risk of bias [8] (Additional file 1).

### Statistical analysis

A random model meta-analysis was performed using RevMan 5.41 (Cochrane Collaboration, London, UK). Using the mean difference (MD), the CFR results were compared between patients with cardiovascular disease (stable CAD, HCM, DCM), and a healthy control HR meta-analysis was performed of stable CAD using the general inverse variance method. Heterogeneity was indicated by I^2^, where 0% indicated no heterogeneity and 100% indicated strong heterogeneity (p < 0.05), which was considered statistically significant.

## Results

### Study characteristics

**Fig. 1:**
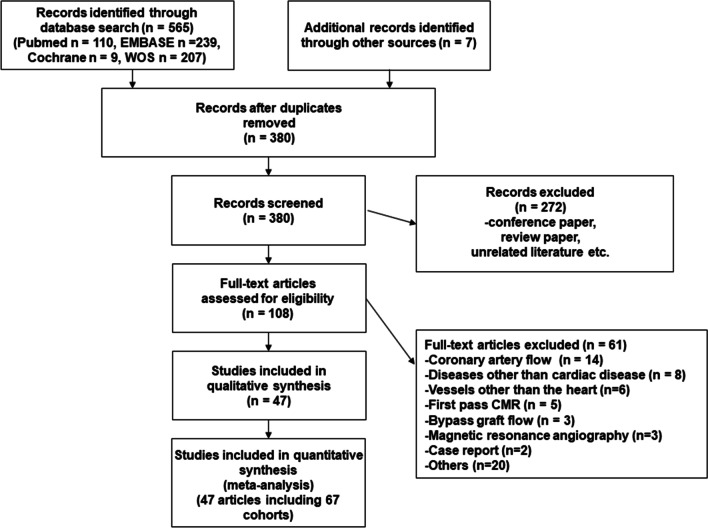
Preferred reporting items for systematic reviews and meta-analyses flow diagram

Of the 565 candidate studies, 47 were deemed eligible for inclusion [7, 9–55] (Fig. 1). Fourteen studies presented two populations each [9, 11, 15–17, 23, 25, 27, 29, 31, 32, 34, 47, 49], while two studies presented four populations each [30, 35]. Therefore, a total of 67 independent populations were included. Among the 47 studies, 22 were from Japan [19–21, 23–37, 42, 44, 45, 47], eight from the United States [7, 14, 22, 40, 41, 48, 51, 55], five from Sweden [12, 16, 17, 53, 54], three from Germany and Sweden [11, 15, 46], two from Finland [38, 39], and one each from Australia [18], France [43], Italy [9], Norway [13], Turkey [10], the United Kingdom [49], Switzerland [7], and the Netherlands [50]. Year of publication ranged from 1992 to 2022. In 35 studies, 1.5T CMR systems were used [7, 9–12, 15–17, 19–21, 24–32, 34–40, 42, 46–49, 51, 52, 54]; in nine studies, 3T CMR systems were used [13, 14, 18, 22, 33, 41, 43, 53, 55]; in one study, both 1.5 and 3T CMR units were used [44]; and in one study, a 0.6T CMR system was used [50]. Information on pharmacological stress was extracted from 41 studies. Four studies used the cold pressor test [21, 33, 41, 43], while two studies did not perform any stress testing [9, 50]. Because CPT detects endothelial function–dependent increases in blood flow that differ significantly from pharmacological stress, only resting blood flow was extracted and integrated from the four CPT studies [21, 33, 41, 43]. A total of 28 (68%) studies used adenosine triphosphate infusion, 10 (24%) used dipyridamole, and three (8%) used the regadenoson. However, no study has employed exercise-stress testing. The median value of velocity encoding (VENC) was set at 50 cm/s at rest (range, 40–200 cm/s). Most studies used the same VENC during rest and stress. In six studies, different VENC were used during pharmacological stress. In four studies [10, 12, 40, 48], the VENC during stress was 200 cm/s. In the study by Gyllenhammar et al. [16], the VENC was 120 cm/s. According to Moro et al. [43], the VENC was 150 cm/s. Thirteen studies performed phase-offset correction using adjacent myocardial tissue [21, 28–31, 33, 35–39, 47, 55], and one study used static tissue regions in the chest wall [55]. The standard imaging parameters and analytical images related to phase-contrast cine CMR are presented (Additional files 1, 2).

### CFR of patients with cardiovascular diseases versus controls

**Fig. 2:**
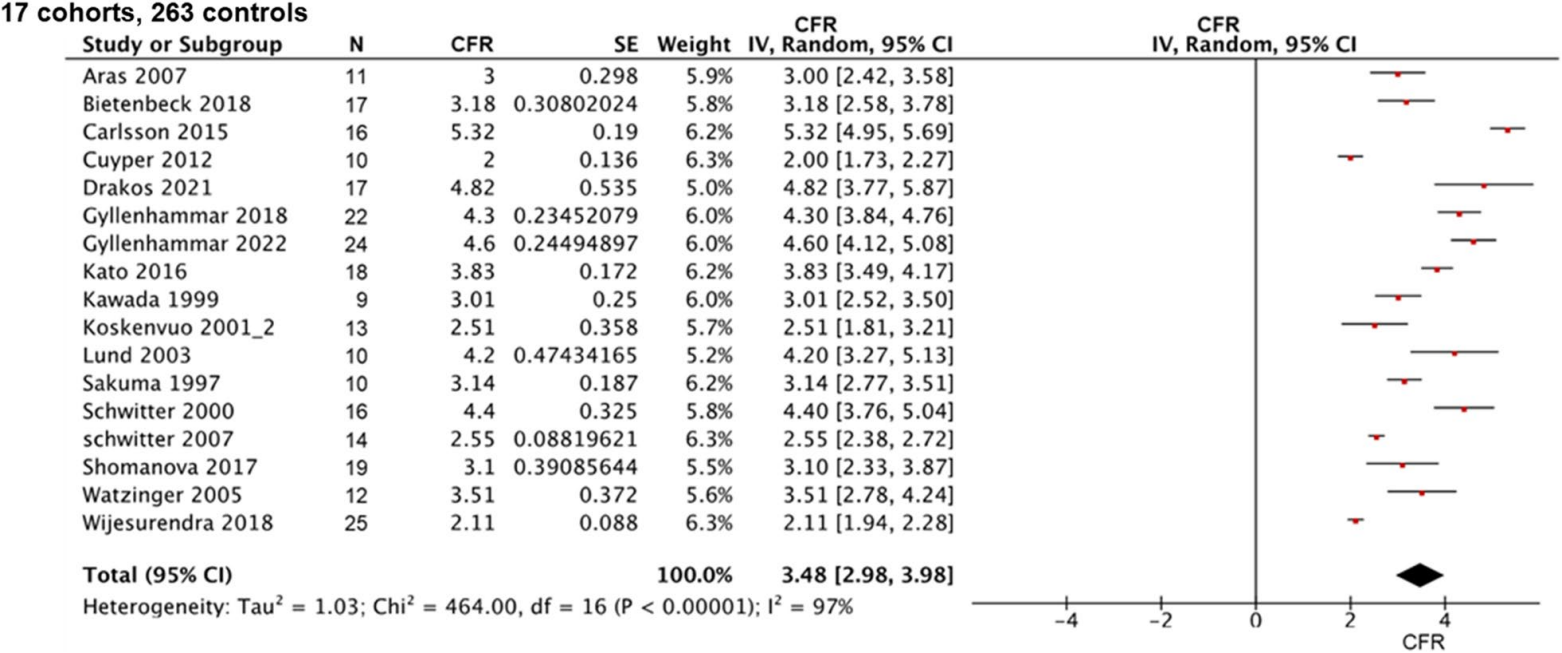
Coronary flow reserve among control subjects. *CFR* coronary flow reserve

**Fig. 3:**
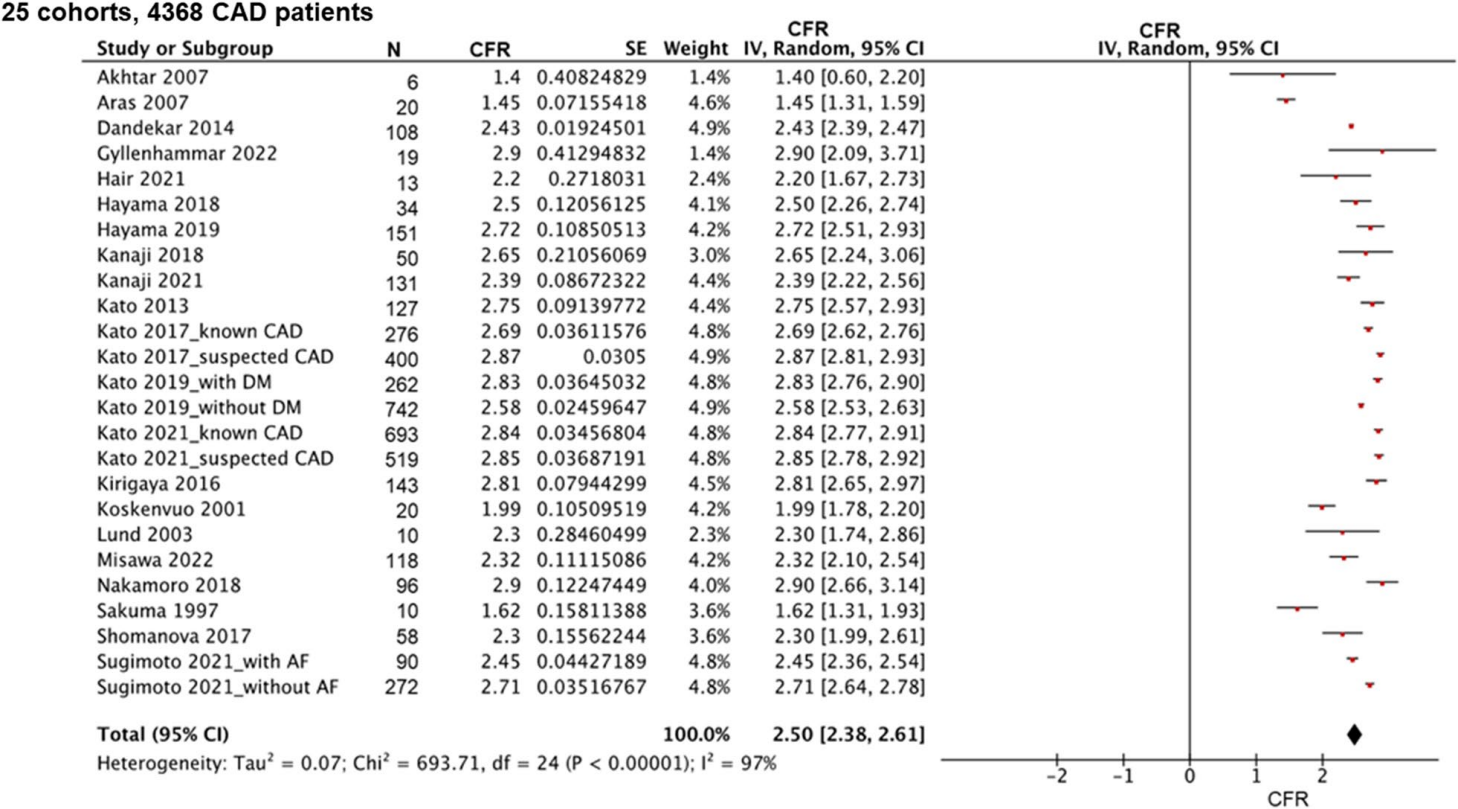
Coronary flow reserve among patients with stable CAD. *CAD* coronary artery disease, *CFR* coronary flow reserve

**Fig. 4:**
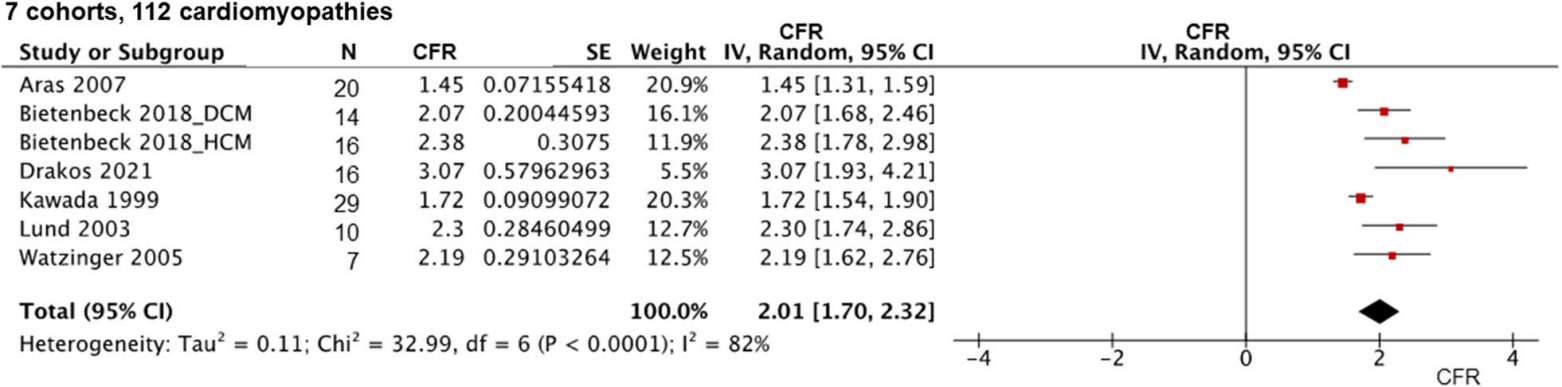
Coronary flow reserve among patients with cardiomyopathies. *CFR* coronary flow reserve, *MBF* myocardial blood flow

**Fig. 5:**
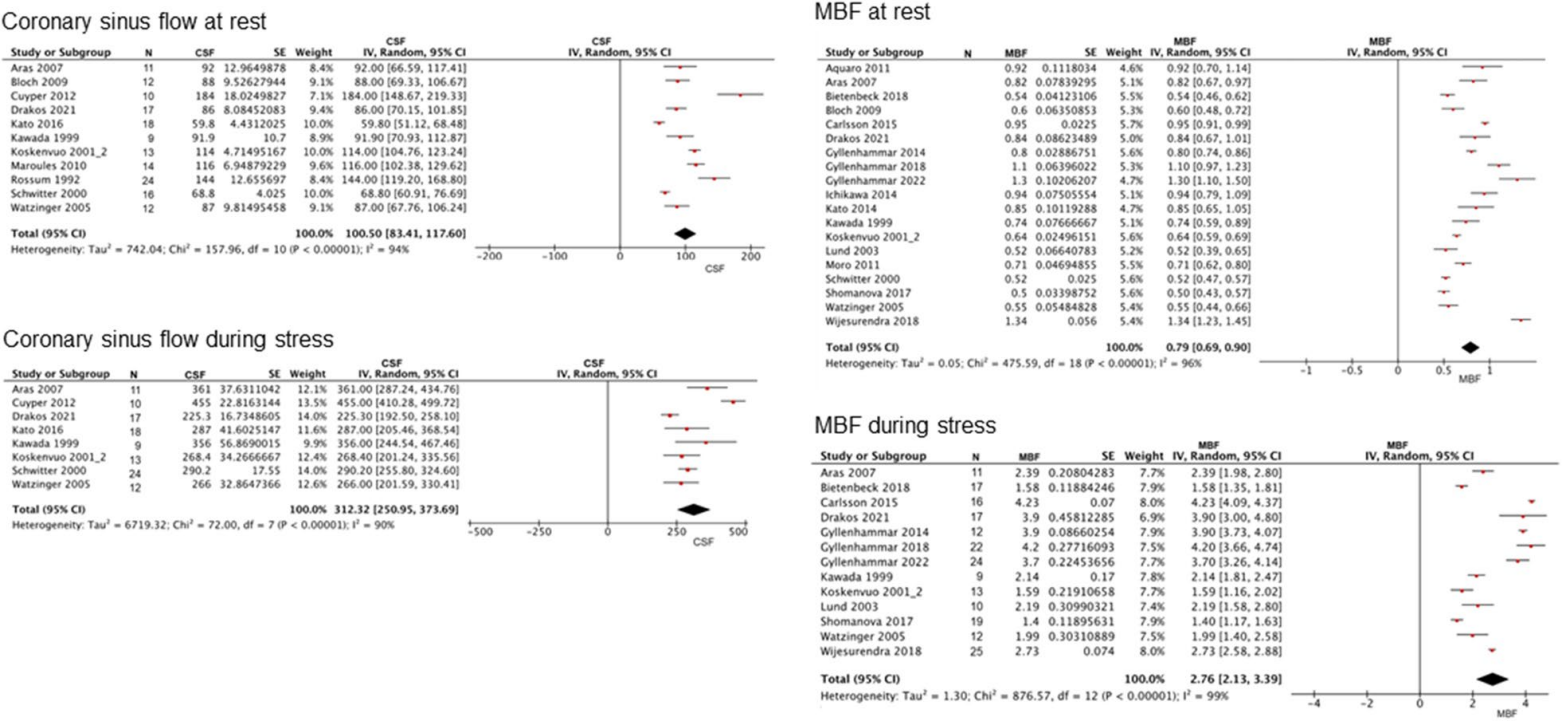
Coronary sinus flow and myocardial blood flow among control subjects. *MBF* myocardial blood flow

**Fig. 6:**
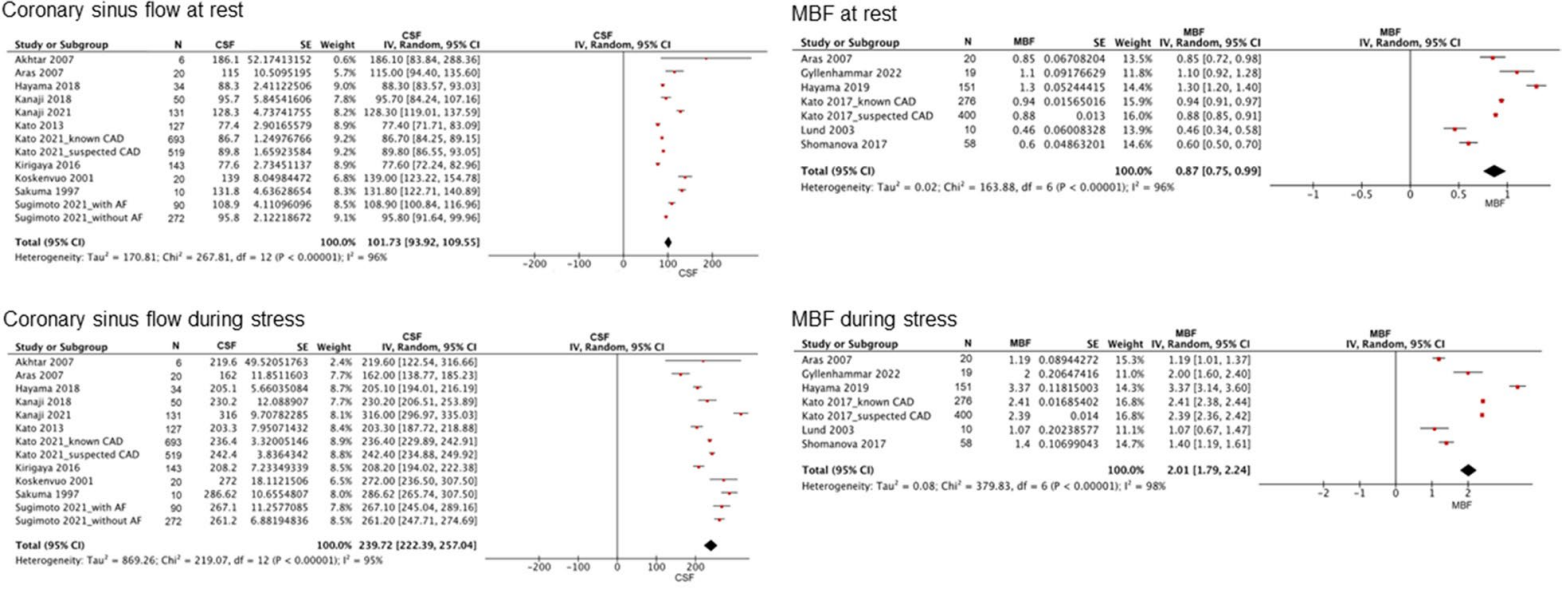
Coronary sinus flow and myocardial blood flow among patients with stable CAD. *CAD* coronary artery disease, *MBF* myocardial blood flow

**Fig. 7:**
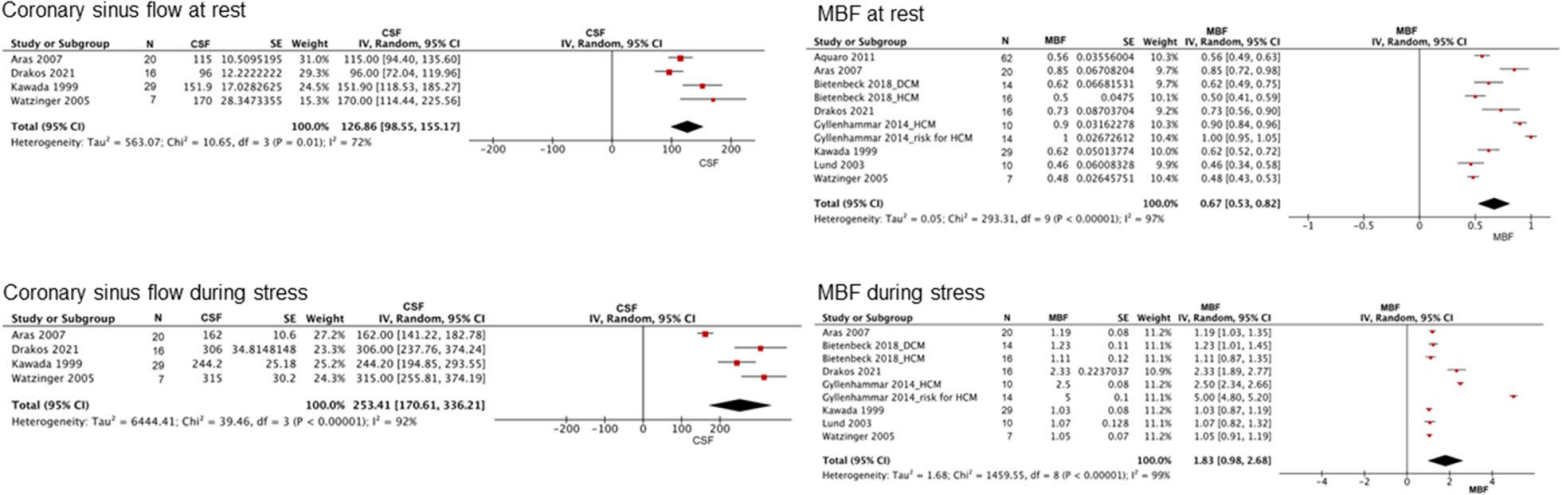
Coronary sinus flow and myocardial blood flow in cardiomyopathies. *MBF* myocardial blood flow

Among the control subjects, the pooled CFR from the meta-analysis was 3.48 (range, 2.98–3.98; I^2^ = 97%; p for heterogeneity < 0.001) (Fig. 2). The estimated CFR was 2.50 (range, 2.38–2.61; I^2^ = 97%; p for heterogeneity < 0.001) for patients with stable CAD (Fig. 3) and 2.01 (range, 1.70–2.32; I^2^ = 82%; p for heterogeneity < 0.001) for those with cardiomyopathies (HCM and DCM) (Fig. 4). Coronary sinus blood flow and MBF values are summarized in Figs. 5, 6 and 7. Among the control subjects, mean coronary sinus blood flow was 100 mL/min (range, 83–118 mL/min) at rest and 312 mL/min (range, 251–374 mL/min) during stress, while the mean MBF was 0.79 mL/min/g (range, 0.69–0.90 mL/min/g) at rest and 2.8 mL/min/g (range, 2.13–3.39 mL/min/g) during stress (Fig. 5).

Among the stable CAD patients, mean coronary sinus blood flow was 102 mL/min (range, 94–110 mL/min) at rest and 240 mL/min (range, 222–257 mL/min) during stress, while the MBF was 0.9 mL/min/g (range, 0.8–1.0 mL/min/g) at rest and 2.0 mL/min/g (range, 1.8–2.2 mL/min/g) during stress (Fig. 6).

**Fig. 8:**
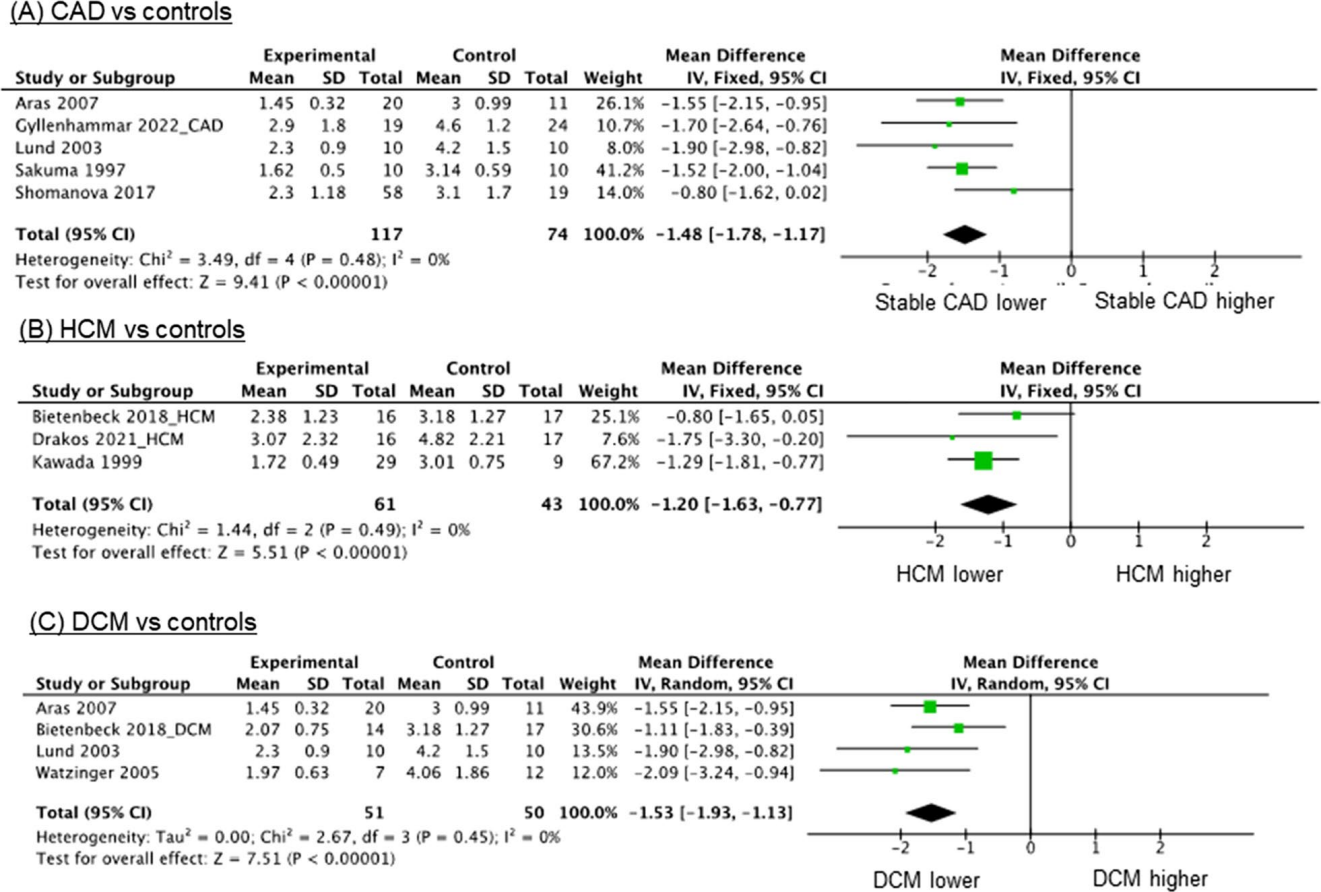
Comparison of CFR between cardiovascular diseases and controls. **A**–**C** Meta-analysis showed that CFR was significantly decreased in various cardiovascular diseases, such as CAD, HCM, and DCM, compared with control subjects. *CAD* coronary artery disease, *CFR* coronary flow reserve, *DCM* dilated cardiomyopathy, *HCM* hypertrophic cardiomyopathy

Among the patients with cardiomyopathies, the mean coronary sinus blood flow was 127 mL/min (range, 99–155 mL/min) at rest and 253 mL/min (range, 171–336 mL/min) during stress, while the mean MBF was 0.7 mL/min/g (range, 0.5–0.8 mL/min/g) at rest and 1.8 mL/min/g (range, 1.0–2.7 mL/min/g) during stress (Fig. 7). The meta-analysis showed that the mean CFR was significantly reduced among the patients with stable CAD (MD = -1.48; 95% CI, -1.78 to -1.17; p < 0.001; I^2^ = 0%; p for heterogeneity = 0.48) (Fig. 8A), HCM (MD = -1.20; 95% CI, − 1.63 to − 0.77; p < 0.001; I^2^ = 0%; p for heterogeneity = 0.49) (Fig. 8B), and DCM (MD = -1.53; 95% CI, − 1.93 to − 1.13; p < 0.001; I^2^ = 0%; p for heterogeneity = 0.45) (Fig. 8C) compared to the control subjects.

### Association between CFR and MACE

**Fig. 9:**
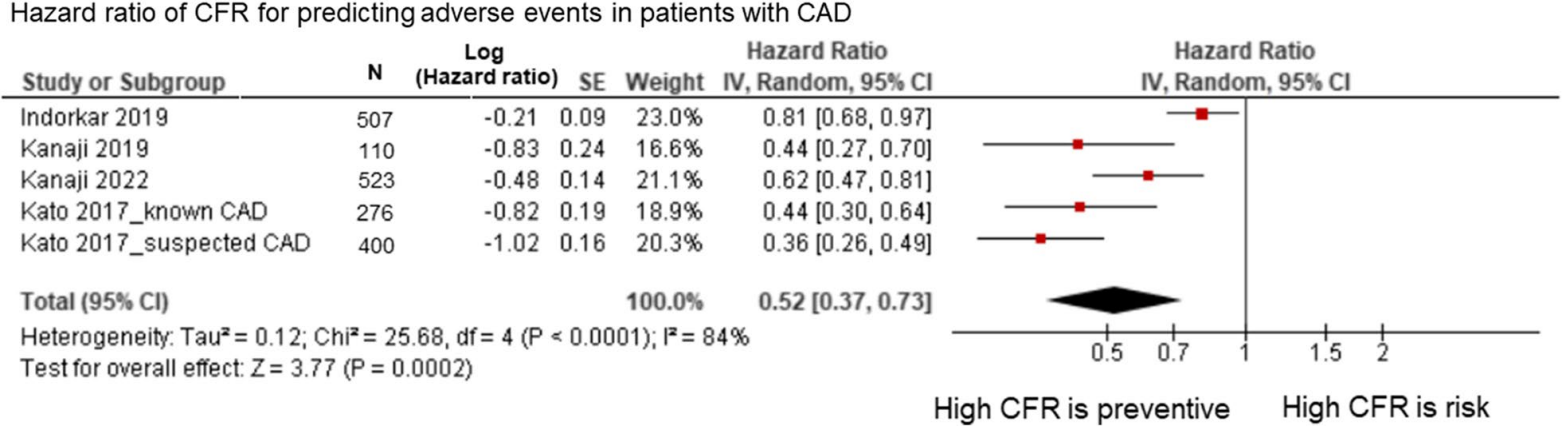
Forest plot of the hazard ratio of CFR among patients with CAD. The meta-analysis revealed that a higher CMR-derived CFR was significantly associated with a lower rate of MACE (HR = 0.52 per unit increase; 95% CI, 0.37–0.73; p < 0.001; I^2^ = 84%, p for heterogeneity < 0.001)**.** *CAD* coronary artery disease, *CFR* coronary flow reserve, *CI* confidence interval, *CMR* cardiac magnetic resonance, *MACE* major adverse cardiac events

Four studies showed outcomes data for patients with stable CAD [22, 27, 30, 35] (Table 1). All studies defined the outcome as a composite endpoint (MACE). Indorkar et al. [22] defined MACE as death, nonfatal myocardial infarction (MI), heart failure hospitalization, sustained ventricular tachycardia, and late revascularization. Kanaji et al. [25] defined MACE as all-cause death, nonfatal MI, hospitalization for congestive heart failure, and stroke. In another study, Kanaji et al. [27] defined MACE as cardiac death, MI, clinically driven remote revascularization (> 3 months after the index percutaneous coronary intervention), or hospitalization for heart failure (HF). Kato et al. [35] defined MACE as cardiovascular death, acute MI, unstable angina, HF hospitalization, or ventricular tachyarrhythmia necessitating defibrillation. A meta-analysis revealed that a higher CMR-derived CFR was significantly associated with a lower MACE rate (HR = 0.52 per unit increase; 95% CI, 0.37–0.73; p < 0.001; I^2^ = 84%; p for heterogeneity < 0.001) (Fig. 9).

**Table 1:** Hazard ratio of CFR for predicting MACE

Study	Variable	Outcome	HR (95% CI)
Kanaji 2022 [25]	Corrected g-CFR (per unit increase)	MACEs: all-cause death, nonfatal myocardial infarction (MI), hospitalization for congestive heart failure, and stroke	0.62 (0.47–0.82)
Kanaji 2019 [27]	Corrected CSF reserve (per unit increase)	MACE: cardiac death, MI, clinically driven remote (> 3 months after the index PCI) revascularization, or hospitalization for congestive heart failure (HF)	0.434 (0.270–0.699)
Indorkar 2019 [22]	CFR (per unit increase)	MACE: death, nonfatal myocardial infarction, heart failure hospitalization, sustained ventricular tachycardia, and late revascularization	0.808 (95% CI: 0.677–0.964 This model uses LGE size and ischemia extent as continuous variables
Kato 2017_known CAD [35]	CFR (univariable), (per unit increase)	MACE: cardiovascular death, acute MI, unstable angina, hospitalization for heart failure or ventricular tachyarrhythmia necessitating defibrillation	0.44 (0.30–0.64)
Kato 2017_suspected CAD [35]	CFR (univariable), (per unit increase)	MACE: cardiovascular death, acute MI, unstable angina, hospitalization for heart failure or ventricular tachyarrhythmia necessitating defibrillation	0.36 (0.26–0.49)

*CFR* coronary flow reserve, *CSF* coronary sinus flow, *g-CFR* global-coronary flow reserve, *MACE* major adverse cardiac events, *LGE* late gadolinium-enhancement, *MI* myocardial infarction, *PCI* percutaneous coronary intervention

## Discussion

The main findings of this study are as follows: (1) CMR-derived CFR was substantially decreased in patients with cardiovascular diseases versus control subjects; and (2) a decreased CFR was associated with a higher rate of MACE among patients with stable CAD. These results suggest that CMR-derived CFR might be applicable as a non-invasive marker to identify abnormal microvascular function in patients with various cardiovascular diseases.

### Importance of microvascular function and CFR assessment by PET imaging

The epicardial coronary arteries (diameter > 400 μm) are conductance vessels, whereas the pre-arterioles (diameter 100–400 μm) and intramural arterioles (diameter < 100 μm) are resistance vessels [5]. Myocardial flow distribution is mainly regulated by small vessels such as the pre-arterioles or arterioles. In MVD, several structural and functional abnormalities occur in these small vessels, such as microvascular remodeling and endothelial dysfunction [56]. These abnormalities are related to attenuated vasodilator response of the smooth muscle cells of vessels resistive to adenosine and dipyridamole associated with impaired CFR. CFR is a functional measure of the epicardial coronary artery and the coronary microcirculation. In the absence of epicardial stenotic disease, a decreased CFR can be a surrogate marker of coronary microvascular dysfunction [57]. PET is the most firmly established non-invasive quantitative assessment method for MBF and CFR. Recent data have demonstrated that the prevalence of a CFR impairment is higher than expected in patients with known or suspected CAD [5]. Moreover, the predictive value of PET-derived CFR is well established [6]. Based on robust clinical evidence, the United States Food and Drug Administration approved the application of ^82^Rubidium and ^13^*N*-Ammonia PET for assessing CFR [5]. However, the utility of PET for evaluating myocardial perfusion has not yet been established.

### CFR assessment using phase-contrast cine MRI of the coronary sinus

Despite the rich clinical evidence of PET imaging for assessing CFR, it features some limitations, such as radiation exposure, limited accessibility, and high cost. Phase-contrast cine CMR of the coronary sinus is also used to quantify CFR. This method has an absolute advantage over PET in that it does not involve radiation exposure. The coronary sinus theoretically drains approximately 96% of the total LV myocardial blood flow [58]; therefore, the LV myocardial blood flow can be calculated by measurement of coronary sinus blood flow. Previous studies reported that phase-contrast cine CMR-derived coronary sinus blood flow correlates well with myocardial blood flow by PET [7]. This evidence demonstrates the high accuracy of CMR-derived CFR.

To date, clinical evidence of CMR-derived CFR is relatively limited. Therefore, here we performed a meta-analysis to determine its clinical relevance. Compared with controls, we found a significant decrease in CFR in various cardiovascular diseases, such as CAD and cardiomyopathies (Figs. 2, 3 and 4). Moreover, CMR-derived CFR can provide prognostic information for patients with CAD. Regarding its technical aspects, setting VENC and phase-offset correction is essential to an accurate evaluation of the blood flow within the coronary sinus. The median VENC was 50 cm/s at rest (40–200 cm/s). Suboptimal VENC settings can lead to aliasing, which can lead to blood flow under- or overestimations. A 40 cm/s VENC at adenosine loading is generally not considered sufficient to increase coronary flow by a factor of 3 to 4, especially during stress; therefore, the VENC should be increased appropriately.

Regarding phase offset correction, 13 studies used adjacent myocardial tissue and one used static tissue regions at the chest wall. Other studies did not perform phase offset correction. Some studies reported the importance of the impact of phase offset correction but did not explain why phase offset correction was not performed. In addition, because of our limited technical knowledge of phase offset correction, we could not adequately determine its importance (whether it is absolutely necessary) in this study. Further research on this topic is required. Evaluation of the effect of medical therapy, such as angiotensin-converting enzyme inhibitors/angiotensin receptor blockers and statins on CFR, is of interest. A recent meta-analysis showed the impact of medical therapies on CFR [59]. However, CMR data were not included thus, further studies are warranted to assess the utility of phase-contrast CMR in the serial impact of medical therapies on CFR.

We believe that our data will contribute to the accurate assessment of CFR using phase-contrast cine CMR of the coronary sinus. However, the relationship between the CMR-derived CFR and stress-perfusion CMR should be recognized. Although this study shows great potential for CMR-derived CFR, the mainstream method remains stress-perfusion CMR. CMR-derived CFR should be performed within a protocol featuring stress-perfusion CMR. However, CMR-CFR has the potential to assess the global microcirculation, which is difficult to assess using stress-perfusion CMR, and may add new CMR value to the diagnosis of INOCA in the post-ischemia era [60].

## Limitations

Our study has several limitations. First, evidence of the prognostic value of CMR-derived CFR mainly targets patients with known or suspected CAD. However, this should be recognized as a limitation because event definitions differed among the studies integrated here. The predictive value of future adverse events for other cardiovascular diseases remains unknown. Second, CMR-derived CFR can assess global and not regional CFR. It is important to remember that stress-perfusion CMR is the mainstream examination method for diagnosing ischemia, whereas phase-contrast cine CMR is an ancillary examination. For example, regional ischemia can occur in patients with a reduced or normal CFR, and stress-perfusion CMR is important. Recent American Heart Association/American College of Cardiology guidelines also recommend a combination of ischemia and CFR for the evaluation of INOCA and do not recommend using CFR alone for risk stratification. Third, in the present study, data were integrated with and without phase-offset correction. This should be recognized as a limitation because the possibility of a significant impact of phase-offset correction on MBF values cannot be denied. Fourth, it is important to reiterate that CMR-derived CFR is not a microcirculation-specific index but rather a composite index of epicardial and endocardial coronary perfusion. That is, the studies included in this review did not necessarily exclude epicardial coronary artery stenosis by invasive X-ray coronary angiography or coronary computed tomography; therefore, caution must be exercised in interpreting the results. Fifth, several studies used CPT as a stress method, but we integrated only resting status data in the present meta-analysis since CPT exhibits an endothelial function–dependent blood flow-increasing response, creating the need to clearly distinguish it from endothelial function–independent blood flow-increasing responses to other agents (e.g., adenosine, regadenoson, adenosine triphosphate, and dipyridamole).

## Conclusions

Phase-contrast cine CMR-derived CFR was significantly decreased in stable CAD and cardiomyopathy, while a decreased CFR was associated with the occurrence of adverse cardiovascular events in patients with stable CAD. These results suggest that CMR-derived CFR has great potential for the pathological evaluation of stable CAD, cardiomyopathy, and risk stratification in CAD.

## Supplementary Information

**Additional file 1.** Search formulas; Newcastle–Ottawa quality assessment scale case control studies; Representative Imaging parameter for phase-contrast cine MRI of the coronary sinus.

**Additional file 2.** Flow measurement of coronary sinus using phase contrast images.

## Data Availability

The datasets analyzed in the current study are available from the corresponding author upon reasonable request.

## Abbreviations

CAD: Coronary artery disease
CFR: Coronary flow reserve
CI: Confidence interval
CMR: Cardiovascular magnetic resonance
CSF: Coronary sinus flow
DCM: Dilated cardiomyopathy
HCM: Hypertrophic cardiomyopathy
HF: Heart failure
HR: Hazard ratio
INOCA: Ischemia with no obstructive coronary artery disease
LGE: Late gadolinium enhancement
LV: Left ventricle/left ventricular
MACE: Major adverse cardiac events
MBF: Myocardial blood flow
MD: Mean difference
MI: Myocardial infarction
MVD: Microvascular dysfunction
PET: Positron emission tomography
VENC: Velocity encoding
