# Supplementary material for: Coronary flow reserve evaluated by phase-contrast cine cardiovascular magnetic resonance imaging of coronary sinus: a meta-analysis
Source: J Cardiovasc Magn Reson. 2023 Feb 20;25:11. doi: 10.1186/s12968-023-00912-5 (PMC9940433; doi:10.1186/s12968-023-00912-5)
Supplement: Supplementary file 1 — Additional file 1. Search formulas; Newcastle–Ottawa quality assessment scale case control studies; Representative Imaging parameter for phase-contrast cine MRI of the coronary sinus. [file 12968_2023_912_MOESM1_ESM.docx]

**Additional file 1**

*PubMed　110*

*(coronary flow reserve [title] OR CFR [title] OR myocardial perfusion reserve [title] OR MPR [title]) AND (magnetic resonance imaging [title] OR MRI [title] OR cardiac magnetic resonance [title] OR CMR [title]) AND (phase contrast [title] OR phase contrast cine MRI [title] OR PC cine MRI [title] OR coronary sinus blood flow [title])*

*WOS 207*

*#1 TI=(coronary flow reserve [title] OR CFR [title] OR myocardial perfusion reserve [title] OR MPR [title])*

*#2 TS=(magnetic resonance imaging [title] OR MRI [title] OR cardiac magnetic resonance [title] OR CMR [title])*

*#3 TS=(phase contrast [title] OR phase contrast cine MRI [title] OR PC cine MRI [title] OR coronary sinus blood flow [title])*

*#4 #1 AND #2 AND #3*

*Cochrane 9*

*#1 coronary flow reserve:ti OR CFR:ti OR myocardial perfusion reserve:ti OR MPR:ti*

*#2 magnetic resonance imaging OR MRI OR cardiac magnetic resonance OR CMR*

*#3 phase contrast OR phase contrast cine MRI OR PC cine MRI OR coronary sinus OR coronary sinus blood flow*

*#4 #1 AND #2 AND #3*

*EMBASE 239*

*QUICK SEARCH:*

*TITLE: (coronary flow reserve OR CFR OR myocardial perfusion reserve OR MPR) AND*

*(magnetic resonance imaging OR MRI OR cardiac magnetic resonance OR CMR) AND*

*(phase contrast OR phase contrast cine MRI OR PC cine MRI OR coronary sinus OR coronary sinus blood flow)*

NEWCASTLE - OTTAWA QUALITY ASSESSMENT SCALE CASE CONTROL STUDIES

| **Study** | **Selection**  **(Max=4)** | **Comparability**  **(Max=2)** | **Exposure**  **(Max=3)** | **Total Score** |
| --- | --- | --- | --- | --- |
| Bietenbeck 2018 | 4 | 1 | 3 | 8 |
| Carlsson 2015 | 4 | 2 | 3 | 9 |
| Cuypers 2012 | 4 | 2 | 3 | 9 |
| Dandekar 2014 | 4 | 0 | 2 | 6 |
| Drakos 2021 | 4 | 2 | 3 | 9 |
| Gyllenhammar 2022 | 4 | 1 | 3 | 8 |
| Gyllenhammar 2014 | 4 | 1 | 3 | 8 |
| Hayama 2018 | 4 | 0 | 3 | 7 |
| Ichikawa 2014 | 4 | 1 | 3 | 8 |
| Kanaji 2020 | 4 | 0 | 2 | 6 |
| Kanaji 2021 | 4 | 0 | 3 | 7 |
| Kanaji 2022 | 4 | 0 | 3 | 7 |
| Kanaji 2018 | 4 | 0 | 3 | 7 |
| Kanaji 2019 | 4 | 0 | 3 | 7 |
| Kato 2013 | 4 | 0 | 3 | 7 |
| Kato 2020 | 4 | 0 | 3 | 7 |
| Kato 2021 | 4 | 0 | 3 | 7 |
| Kato 2021 | 4 | 0 | 2 | 6 |
| Kato 2016 | 4 | 2 | 3 | 9 |
| Koskenvuo 2001 | 4 | 0 | 3 | 7 |
| Misawa 2022 | 4 | 0 | 3 | 7 |
| Moro 2011 | 4 | 0 | 3 | 7 |
| Nakamori 2018 | 4 | 0 | 3 | 7 |
| Shomanova 2017 | 4 | 1 | 3 | 8 |
| Sugimoto 2021 | 4 | 0 | 3 | 7 |
| Watzinger 2005 | 4 | 1 | 3 | 8 |
| Wijesurendra 2018 | 4 | 2 | 3 | 9 |
| Sakuma 1997 | 4 | 1 | 2 | 7 |
| Kawada 1999 | 4 | 1 | 2 | 7 |
| Lund 2003 | 4 | 2 | 2 | 8 |
| Aquaro 2011 | 4 | 1 | 3 | 8 |
| Halabi 2021 | 4 | 0 | 3 | 7 |
| Hayama 2019 | 4 | 0 | 3 | 7 |
| Indorkar 2019 | 4 | 0 | 3 | 7 |
| Kato 2019 | 4 | 0 | 2 | 6 |
| Kato 2014 | 4 | 2 | 2 | 8 |
| Kato 2017 | 4 | 0 | 2 | 6 |
| Kirigaya 2016 | 4 | 0 | 3 | 7 |
| Maroules 2010 | 4 | 0 | 3 | 7 |
| Aras 2007 | 4 | 2 | 3 | 9 |
| Rossum 1992 | 4 | 1 | 3 | 8 |
| Akhtar 2007 | 4 | 0 | 2 | 6 |
| Schwitter 2007 | 4 | 2 | 2 | 8 |
| Bloch 2009 | 4 | 2 | 3 | 9 |
| Gyllenhammar 2018 | 4 | 2 | 2 | 8 |
| Schwitter 2000 | 4 | 2 | 3 | 9 |
| Hair 2021 | 4 | 0 | 3 | 7 |

**Representative Imaging parameter for phase-contrast cine MRI of the coronary sinus**

| Vector-electrocardiogram-triggered gradient echo sequence |
| --- |
| repetition time: 7.3 ms |
| echo time: 4.4 ms |
| flip angle:10 |
| field of view: 380x228 mm |
| acquisition matrix:160x160 |
| reconstruction matrix: 256x256 |
| reconstruction resolution: 1.48x1.48 mm |
| number of phases per cardiac cycle: 20 |
| velocity encoding: 50 cm/s at rest, 100 cm/s during stress |
| slice thickness: 6mm |
