# Supplementary figures and images for: Coronary flow reserve evaluated by phase-contrast cine cardiovascular magnetic resonance imaging of coronary sinus: a meta-analysis
Source: J Cardiovasc Magn Reson. 2023 Feb 20;25:11. doi: 10.1186/s12968-023-00912-5 (PMC9940433; doi:10.1186/s12968-023-00912-5)

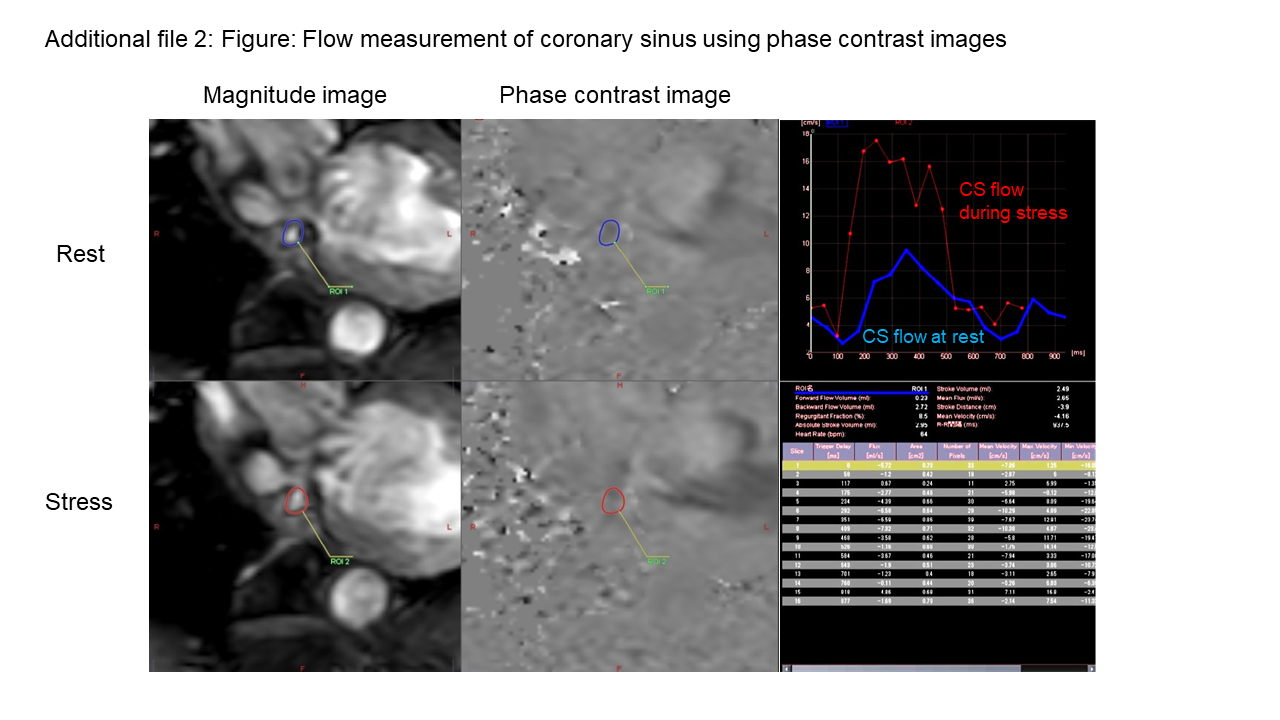

Supplement: Supplementary file 2 — Additional file 2. Flow measurement of coronary sinus using phase contrast images. [file 12968_2023_912_MOESM2_ESM.tif]
